# Observational *versus* antibiotic treatment for uncomplicated diverticulitis: an individual‐patient data meta‐analysis

**DOI:** 10.1002/bjs.11465

**Published:** 2020-02-19

**Authors:** S. T. van Dijk, A. Chabok, M. G. Dijkgraaf, M. A. Boermeester, K. Smedh

**Affiliations:** ^1^ Department of Surgery Academic Medical Centre Amsterdam the Netherlands; ^2^ Department of Clinical Epidemiology, Biostatistics and Bioinformatics Academic Medical Centre Amsterdam the Netherlands; ^3^ Department of Surgery and Centre for Clinical Research Uppsala University, Västmanlands Hospital Västerås Sweden

## Abstract

**Background:**

Two RCTs (AVOD and DIABOLO) demonstrated no difference in recovery or adverse outcomes when antibiotics for acute uncomplicated diverticulitis were omitted. Both trials showed non‐significantly higher rates of complicated diverticulitis and surgery in the non‐antibiotic groups. This meta‐analysis of individual‐patient data aimed to explore adverse outcomes and identify patients at risk who may benefit from antibiotic treatment.

**Methods:**

Individual‐patient data from those with uncomplicated diverticulitis from two RCTs were pooled. Risk factors for adverse outcomes and the effect of observational management were assessed using logistic regression analyses. *P* < 0·025 was considered statistically significant owing to multiple testing adjustment.

**Results:**

In total, 545 patients in the observational group and 564 in the antibiotics group were included. No statistical differences were found in 1‐year follow‐up rates of ongoing diverticulitis (7·2 *versus* 5·0 per cent in observation *versus* antibiotics groups respectively; *P* = 0·062), recurrent diverticulitis (8·6 *versus* 9·6 per cent; *P* = 0·610), complicated diverticulitis (4·0 *versus* 2·1 per cent; *P* = 0·079) and sigmoid resection (5·0 *versus* 2·5 per cent; *P* = 0·214). An initial pain score greater than 7, white blood cell count exceeding 13·5 × 10^9^/l and previous diverticulitis at presentation were risk factors for adverse outcomes. Antibiotic treatment did not prevent adverse outcomes in patients at high risk of adverse events.

**Conclusion:**

Observational management of acute uncomplicated diverticulitis is safe. Some statistical uncertainty remains, depending on the thresholds of clinical relevance, owing to small differences, but no subgroup that would benefit from antibiotic treatment was apparent.

## Graphical abstract

This individual‐patient data meta‐analysis of two RCTs, including 1109 patients, showed that non‐antibiotic management of acute uncomplicated diverticulitis is safe, given the comparable rates of adverse events up to 1 year compared with routine antibiotic treatment. Some statistical uncertainty may remain depending on thresholds of clinical relevance as the sample size of the meta‐analysis was insufficient to detect small differences. However, no patient subgroup could be identified that could potentially benefit from antibiotic treatment.

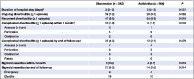

Antibiotics not needed

## Abstract (es)

**Antecedentes:**

Dos ensayos clínicos aleatorizados y controlados (*randomized controlled trials,* RCTc) (AVOD y DIABOLO) no mostraron diferencias en la recuperación ni en los resultados adversos al tratar la diverticulitis aguda no complicada sin antibióticos. Ambos ensayos mostraron tasas más altas, estadísticamente no significativas, de diverticulitis complicada y cirugía en los grupos sin antibióticos. Este metaanálisis de datos individuales de pacientes tuvo como objetivo explorar resultados adversos e identificar pacientes en riesgo que pudieran beneficiarse del tratamiento con antibióticos.

**Métodos:**

Se agruparon los datos individuales de pacientes con diverticulitis no complicada de dos RCT. Los factores de riesgo para los resultados adversos y el efecto del tratamiento observacional (sin antibióticos) se evaluaron mediante un análisis de regresión logística. Una *P* < 0,025 se consideró estadísticamente significativa debido al ajuste de múltiples comparaciones.

**Resultados:**

En total, se incluyeron 545 pacientes en el grupo de observación y 564 pacientes en el grupo de tratamiento antibiótico. No se encontraron diferencias estadísticamente significativas (observación *versus* antibióticos, respectivamente) en el seguimiento a 1 año en los porcentajes de diverticulitis persistente (7,2% *versus* 5,0%; *P* = 0,062), diverticulitis recurrente (8,6% *versus* 9,6%; *P* = 0,610), diverticulitis complicada (4,0% *versus* 2,1%; *P* = 0,079) y resección sigmoidea (5,0% *versus* 2,5%; *P* = 0,214). Una puntuación de dolor inicial > 7, un recuento leucocitario > 13,5 x 10^9^/L y los antecedentes de diverticulitis fueron factores de riesgo para un resultado adverso. El tratamiento con antibióticos no previno los resultados adversos en pacientes con alto riesgo.

**Conclusión:**

El tratamiento de la diverticulitis aguda no complicada basado en la observación es seguro. No obstante, persiste una cierta incertidumbre desde el punto de vista estadístico, dependiendo de los umbrales de relevancia clínica, debido a pequeñas diferencias, pero no fue evidente ningún subgrupo que se pudiera beneficiar del tratamiento con antibióticos.

## Introduction

Acute uncomplicated diverticulitis has been treated routinely with antibiotics for decades, although evidence in favour of antibiotic treatment has always been lacking. Recently two RCTs^1^, ^2^ demonstrated the safety of omitting antibiotics in the treatment of acute uncomplicated diverticulitis up to 1 year of follow‐up. First, the AVOD Antibiotics in Acute Uncomplicated Diverticulitis trial^1^ showed comparable rates of complicated diverticulitis, recurrent diverticulitis and sigmoid resection between observational and antibiotic treatment. Second, the DIABOLO (Diverticulitis: Antibiotics or Close Observation) trial^2^ found no significant differences regarding time to recovery, complicated diverticulitis, recurrent diverticulitis and sigmoid resection. However, both RCTs reported somewhat – by and large non‐significant – higher rates of complicated diverticulitis and sigmoid resections in the non‐antibiotic group. As neither of the studies was powered to analyse these secondary outcomes, uncertainty remained whether these small differences could potentially be true causal associations, the consequence of patient selection or findings by chance in accordance with statistical non‐significance. A regular meta‐analysis of the two RCTs would not be able to account for different follow‐up times and different outcome definitions that were provided in the results papers of these studies. In contrast, a meta‐analysis of individual‐patient data can not only account for these differences but additionally provide an opportunity to use the increased sample size of both trials combined. The aim was to identify patients at risk of complications who may benefit from antibiotic treatment.

## Methods

### Study design and patient population

Individual‐patient data from two open‐label RCTs (AVOD and DIABOLO) were pooled in the present study. The AVOD trial^1^ was conducted in 11 hospitals in Sweden and Iceland during 2003–2010. The DIABOLO trial^2^ was carried out in 22 hospitals in the Netherlands during 2010–2012. Both trials included only patients with left‐sided, CT‐proven acute uncomplicated diverticulitis, and excluded immunocompromised patients, pregnant women and patients with signs of sepsis. Patients with small pericolic abscesses (Hinchey stage 1b^3^) from the DIABOLO trial were excluded from the present study, because the AVOD trial included only patients without abscesses and the number of patients with Hinchey 1b disease in the DIABOLO trial was small (42 of 528). Broad‐spectrum antibiotics were used in both trials. Those in AVOD received antibiotics intravenously or orally for 7 days, whereas patients in DIABOLO were treated for a total of 10 days, with intravenous administration for at least 48 h. In the AVOD trial, all patients were admitted, then discharged based on the assessment of the attending surgeon in both study groups. In the DIABOLO trial, patients in the observational group could be treated as outpatients when predefined criteria were met, whereas those in the antibiotics group were all admitted on the premise that treatment was started intravenously, and were considered for outpatient treatment only after 48 h. *Table* 1 shows a summary of study characteristics of the trials.

**Table 1:** Summary of characteristics of included studies

	DIABOLO	AVOD
**Study design**	Open‐label RCT	Open‐label RCT
**Study setting**	22 hospitals in the Netherlands during 2010–2012	11 hospitals in Sweden and Iceland during 2003–2010
**Patients**	528 patients (262 in observational group and 266 in antibiotics group)	623 patients (309 in observational group and 314 in antibiotics group)
**Inclusion criteria**	CT‐proven, left‐sided acute uncomplicated (Hinchey stage 1a and 1b) diverticulitis	CT‐proven, left‐sided, acute uncomplicated diverticulitis (without any sign of complications such as abscess, free air or fistula), temperature ≥ 38°C at admission or during the 12 h before admission, raised WBC and CRP level, or increased WBC if short history
**Exclusion criteria**	Previous diverticulitis, pregnancy, inflammatory bowel disease, ASA fitness grade > III, immunocompromised, clinical suspicion of bacteraemia (sepsis^4^)	Pregnancy, immunosuppressive therapy, high fever, affected general condition, peritonitis or sepsis
**Intervention**		
Antibiotic treatment	10‐day course of amoxicillin clavulanic acid, with intravenous administration for at least 48 h, switched to oral administration if tolerated; admission of all patients on the premise that treatment was started intravenously; discharge when meeting criteria: toleration of a normal diet, temperature < 38°C, pain score < 4, capable of self‐support at same level as before illness, and patient acceptance	7‐day course of intravenous or oral broad‐spectrum antibiotics according to the participating centres' routines; admission of all patients; discharge based on assessment of attending surgeon with improvement in clinical status, as well as a reduction in WBC and CRP level, and absence of fever
Observational treatment	Supportive care; outpatient treatment when criteria for outpatient treatment met, as for the antibiotics group	Supportive care; intravenous fluids only; admission of all patients; discharge when criteria met, as for the antibiotics group
**Outcomes**		
Ongoing diverticulitis	Clinical picture of diverticulitis, within 3 months of randomization, or no recovery between randomization and subsequent diverticulitis	Not recorded separately
Recurrent diverticulitis	Clinical picture of diverticulitis, and interval of at least 3 months since randomization, and recovery during this interval	Clinical picture of diverticulitis demanding readmission to hospital
Complicated diverticulitis	Abscess, perforation, obstruction, fistula or diverticular bleeding	Abscess, perforation, obstruction or fistula
**Follow‐up**	Last follow‐up contact and patient record assessment at 24 months	Last follow‐up contact and patient record assessment after a minimum of 12 months

WBC, white blood cell count; CRP, C‐reactive protein.

### Outcomes and follow‐up

Outcome measures in the present study were duration of hospital stay, and rates of ongoing diverticulitis, recurrent diverticulitis, complicated diverticulitis and sigmoid resection. Three outcomes were redefined in order to create definitions as homogeneous as possible: ongoing diverticulitis, recurrent diverticulitis and complicated diverticulitis. In the DIABOLO trial, ongoing diverticulitis and recurrent diverticulitis were distinct outcomes as symptoms of acute diverticulitis within 3 months of randomization were considered a prolongation of the initial diverticulitis episode instead of a true recurrent, hence new, episode. As the AVOD trial analysed all disease activity after discharge from hospital as recurrent episodes, all episodes within 3 months after randomization were redefined as ongoing diverticulitis episodes. All subsequent episodes later than 3 months after randomization were considered recurrent episodes. Furthermore, in the AVOD trial, diverticular bleeding was not recorded as complicated diverticulitis. Therefore, cases of diverticular bleeding in the DIABOLO trial were excluded as a complicated diverticulitis event in the present study. However, as diverticular bleeding was recorded as a potential reason for sigmoid resection in the AVOD trial, it was included as a potential reason for sigmoid resection in the present study as well.

Another difference between the studies was the time of last follow‐up: 12 months in AVOD and 24 months in DIABOLO. To analyse results of the studies equally, outcomes were assessed at 12 months of follow‐up. As patients were contacted after a minimum of 12 months in the AVOD trial, most follow‐up contacts took place in the 13th month after randomization. Therefore, results from both trials were assessed up to 13 months after randomization.

### Statistical analysis

Observational and antibiotic treatment were compared following the intention‐to‐treat principle and differences were assessed for superiority. Numbers and percentages were calculated for categorical variables, and median (i.q.r.) for continuous variables as these data were not normally distributed. As the data in this meta‐analysis are clustered by study, comparison between treatment groups was corrected for this clustering. A generalized linear mixed model was fitted for each outcome measure. A logistic regression mixed model was used for dichotomous outcomes. A γ regression distribution was chosen for the only continuous outcome (duration of hospital stay) as the data were right‐skewed. Both treatment allocation and study were entered into the model as fixed effects. This meta‐analysis tested the outcomes of both trials (although some were slightly modified) in addition to the initial analysis of both trials. Therefore, correction for multiple testing was appropriate and a two‐sided *P* < 0·025 was considered statistically significant. For some relevant outcome measures, a *post hoc* power calculation was performed using the results from unequal groups and without continuity correction.

Multivariable logistic regression was used to identify independent risk factors. Variables that were significant or approached significance (*P* < 0·050) in the univariable analyses were entered into the multivariable logistic regression analyses. To provide insight in the effect of clustering of the data, the univariable analyses were repeated with adjustment for study. All multivariable analyses were adjusted for study. To assess the effect of omitting antibiotics on outcomes in patient subgroups and thereby identifying potential subgroups that may benefit from antibiotics, the interactions between observational treatment and each risk factor that met the criteria for entering the multivariable logistic regression analyses were assessed. A multivariable logistic regression model was created for each risk factor, which contained both the main effects of observational treatment and that risk factor, as the interaction effect of these variables. Next, the effect of omitting antibiotics in patient subgroups with multiple risk factors was assessed in a full factorial analysis. This multivariable logistic regression model included the main effects of observational treatment and all risk factor variables that were univariably associated (*P* < 0·050) with that outcome, and all possible interaction (including 2nd‐ and 3rd‐level interactions) effects between observational treatment and the risk factors. Backward selection, with a *P* > 0·050 significance level for removal of predictors, was used to identify potential significant predictors or interactions. These analyses were also corrected for clustering of the data by including study as co‐variable in all models. All risk estimates are expressed as odds ratios (ORs) with 97·5 per cent confidence intervals.

Continuous variables were converted into dichotomous categorical variables, making them easier to interpret and apply in daily practice. The most common cut‐off values in the literature were used to dichotomize certain variables; 30 kg/m^2^ (obesity or no obesity) for BMI, 50 years for age, and 38°C (fever or no fever) for body temperature. For other continuous variables, the optimal cut‐off was determined using receiver operating characteristic (ROC) curves, as the value giving the highest combined sensitivity and specificity; these included pain score on a visual analogue scale (VAS), C‐reactive protein level and white blood cell count (WBC) at presentation with the initial acute diverticulitis episode (at randomization). All analyses were performed using SPSS® version 24.0 (IBM, Armonk, New York, USA). TRIPOD guidelines^5^ for reporting were followed.

## Results

This individual‐patient data meta‐analysis included 1109 patients, 545 in the observational group and 564 in the antibiotics group. Duration of follow‐up was comparable between the groups: 12·3 (i.q.r. 12·1–13·0) months in the observational group and 12·4 (12·1–13·0) months in the antibiotics group. Baseline characteristics were mostly comparable; however, primary diverticulitis was slightly but significantly more common in the antibiotics group (*Table* 2).

**Table 2:** Baseline characteristics of patients according to study group

	Observation (*n* = 545)	Antibiotics (*n* = 564)	*P* §
Age (years)*	58·3 (47·9–65·8)	57·6 (48·5–65·5)	0·827¶
Sex ratio (M : F)	228 : 317	230 : 334	0·721
Co‐morbidity†	170 (31·2)	190 (33·7)	0·375
BMI (kg/m^2^)*	27·4 (24·6–29·7)	27·3 (24·6–30·5)	0·569¶
Body temperature (°C)*	38·0 (37·2–38·3)	38·0 (37·2–38·3)	0·775¶
White blood cell count (× 10^9^/l)*	12·2 (10·3–14·2)	12·4 (10·4–14·3)	0·773¶
C‐reactive protein (mg/l)*	76·0 (44·0–122·8)	86·0 (48·0–130·0)	0·066¶
Primary diverticulitis	408 (74·9)	454 (80·5)	0·024

Values in parentheses are percentages unless indicated otherwise;

* values are median (i.q.r.).

† Includes cardiovascular disease and/or pulmonary disease and/or renal failure and/or diabetes mellitus.

§ χ^2^ test, except

¶ Mann–Whitney *U* test.

### Observational *versus* antibiotic treatment

The duration of stay for the initial hospital admission was slightly but non‐significantly shorter in the observational group compared with the antibiotics group (median 2 *versus* 3 days respectively; *P =* 0·037) (*Table* 3). Some 33 of 1109 patients (3·0 per cent) were treated as outpatients from the start of treatment. The rate of ongoing diverticulitis differed somewhat, but this difference failed to reach statistical significance: 7·2 per cent (39 of 545) in observational group *versus* 5·0 per cent (28 of 564) in the antibiotics group (*P =* 0·062). The rates of recurrent diverticulitis were comparable: 8·6 per cent (47 of 545) *versus* 9·6 per cent (54 of 564) respectively (*P =* 0·610). Complicated diverticulitis and sigmoid resection rates were recorded during the acute disease stage (within 1 month) and at 1‐year follow‐up. Rates of complicated diverticulitis within 1 month were comparable between groups: 1·8 per cent (10 of 545) in the observational group *versus* 1·1 per cent (6 of 564) in antibiotics group (*P =* 0·204); at 1‐year follow‐up they differed somewhat (by 10 patients), but non‐significantly: 4·0 per cent (22 of 545) *versus* 2·1 per cent (12 of 564) respectively (*P =* 0·079). Rates of sigmoid resection were no different between the groups at 1 month (0·6 per cent (3 of 545) *versus* 0·7 per cent (4 of 564); *P =* 0·818) or 1 year (5·0 per cent (27 of 545) *versus* 2·5 per cent (14 of 564); *P =* 0·214).

**Table 3:** Intention‐to‐treat analyses among patients with Hinchey stage 1a acute diverticulitis assigned to an observational or antibiotic treatment strategy

	Observation (*n* = 545)	Antibiotics (*n* = 564)	*P* §
**Duration of hospital stay (days)** *	2 (2–3)	3 (2–3)	0·037¶
**Ongoing diverticulitis (≥ 1 episode)**	39 (7·2)	28 (5·0)	0·062
**Recurrent diverticulitis (≥ 1 episode)**	47 (8·6)	54 (9·6)	0·610
**Complicated diverticulitis (≥ 1 episode) within 1 month** †	10 (1·8)	6 (1·1)	0·204
Abscess (> 5 cm)	3	1	
Perforation	5	5	
Obstruction	2	0	
**Complicated diverticulitis (≥ 1 episode) by end of follow‐up** †	22 (4·0)	12 (2·1)	0·079
Abscess (> 5 cm)	7	4	
Perforation	8	5	
Obstruction	5	3	
Fistula	3	0	
**Sigmoid resection within 1 month**	3 (0·6)	4 (0·7)	0·818
**Sigmoid resection by end of follow‐up**	27 (5·0)	14 (2·5)	0·214
Emergency	8	4	
Elective	19	10	

Values in parentheses are percentages unless indicated otherwise;

* values are median (i.q.r.). Median follow‐up was 12·3 (i.q.r. 12·1–13·0) months overall, 12·3 (12·1–13·0) months in the observational group and 12·4 (12·1–13·0) months in the antibiotics group.

† Patients could have more than one type of complicated diverticulitis. Groups were compared using generalized linear mixed models:

§ logistic regression mixed model, except

¶ gamma regression mixed model.

Although rates of complicated diverticulitis and sigmoid resection at the end of follow‐up were no different statistically, the differences may be considered clinically relevant. The number needed to treat to prevent one case of complicated diverticulitis would be 53 (95 per cent c.i. –40 to 782) and the number needed to treat to prevent one sigmoid resection would be 41 (21 to 411). Because the DIABOLO trial was not powered for analysis of these secondary outcomes and the AVOD trial was only powered for analysis of complicated diverticulitis, a *post hoc* power analysis was performed for these two outcomes based on the combined results in the present study. For the difference in complicated diverticulitis rates, this comparison had a power of 34 per cent. To achieve a power of 80 per cent with an α of 0·05, which is generally considered adequate for intervention trials, a new single study would need a sample size of 2570 patients. For the difference in sigmoid resection rates, this comparison had a power of 48 per cent, and a new single study with a power of 80 per cent and an α of 0·05 would need a sample size of 1811 patients.

### Risk factors and role of antibiotics in prevention of adverse outcomes

Although no statistically significant differences were found between treatment groups, some of the differences in adverse event rates may be considered clinically relevant. If so, the statistical power appeared to be insufficient. Furthermore, even though antibiotic treatment may not be effective for the entire study group, some patients may possibly benefit from such treatment. Therefore, additional analyses were performed to assess the potential role of antibiotics in the prevention of these adverse outcomes. Risk factors for the development of ongoing diverticulitis, complicated diverticulitis and sigmoid resection were assessed. Additionally, to maximize power in the logistic regression analyses, risk factors for ongoing diverticulitis, complicated diverticulitis or undergoing a sigmoid resection were evaluated as a single adverse outcome group (*Table* 4; full results of the univariable logistic regression models are available in *Tables* *S1–S4*, supporting information). A primary episode of diverticulitis instead of recurrent diverticulitis appeared to be protective against ongoing diverticulitis (OR 0·29, 97·5 per cent c.i. 0·13 to 0·66). Risk factors for the development of complicated diverticulitis were a VAS pain score of more than 7 (OR 2·78, 1·18 to 6·54) and WBC higher than 13·5 × 10^9^/l (OR 2·62, 1·11 to 6·18). A pain score of more than 7 was also a risk factor for sigmoid resection (OR 2·32, 1·05 to 5·10). Analyses of all three adverse outcomes as a single adverse event group yielded no additional risk factors; again, a pain score of more than 7 and WBC count higher than 13·5 × 10^9^/l were identified as risk factors, and primary diverticulitis as a protective factor.

**Table 4:** Results of multivariable analyses of risk factors associated with ongoing diverticulitis, complicated diverticulitis, sigmoid resection or all three outcomes combined

	Odds ratio				
	No. of patients at risk	Ongoing diverticulitis	Complicated diverticulitis	Sigmoid resection	Ongoing or complicated diverticulitis, or sigmoid resection
**VAS score at presentation** *					
≤ 7	650		1·00 (reference)	1·00 (reference)	1·00 (reference)
> 7	261		2·78 (1·18, 6·54)	2·32 (1·05, 5·10)	1·98 (1·18, 3·34)
**Temperature at presentation (°C)**					
≤ 38·0	643			1·00 (reference)	
> 38·0	462			0·66 (0·25, 1·70)	
**WBC at presentation (× 10** ^**9**^ **/l)** *					
≤ 13·5	736		1·00 (reference)		1·00 (reference)
> 13·5	370		2·62 (1·11, 6·18)		1·76 (1·05, 2·95)
**Primary diverticulitis**					
No	247	1·00 (reference)			1·00 (reference)
Yes	862	0·29 (0·13, 0·66)			0·33 (0·16, 0·71)

Values in parentheses are 97·5 per cent confidence intervals.

* Cut‐off at optimal sensitivity and specificity according to receiver operating characteristic (ROC) curve analysis. Variables that were assessed univariably but were not entered into the multivariable analyses were: sex, BMI over 30 kg/m^2^, age over 50 years, present co‐morbidity and C‐reactive protein level at presentation. VAS, visual analogue scale; WBC, white blood cell count.

The effect of omitting antibiotics on all risk factors that had been entered into the multivariable analyses was evaluated to assess whether antibiotic treatment could alter the risk in patients susceptible to any adverse outcome and potentially prevent these adverse outcomes in specific patient subgroups. Observational management did not significantly increase the risk of adverse outcomes in any subgroup of patients (*Table* 5). Furthermore, observational treatment failed to influence outcomes in all possible combinations of risk factors in the full factorial analysis. Therefore, no patient subgroup that could potentially benefit from antibiotic treatment was identified.

**Table 5:** Interaction between observational treatment and risk factors that were associated univariably (*P* < 0·050) with one of the outcomes, assessed using multivariable logistic regression analyses

	Odds ratio			
	Ongoing diverticulitis	Complicated diverticulitis	Sigmoid resection	Ongoing or complicated diverticulitis, or sigmoid resection
**VAS score at presentation** *				
≤ 7		1·00 (reference)	1·00 (reference)	1·00 (reference)
> 7		0·20 (0·02, 1·62)	0·24 (0·04, 1·50)	0·94 (0·33, 2·68)
**Temperature at presentation (°C)**				
≤ 38·0			1·00 (reference)	
> 38·0			2·48 (0·34, 17·93)	
**WBC at presentation (× 10** ^**9**^ **/l)** *				
≤ 13·5		1·00 (reference)		1·00 (reference)
> 13·5		0·99 (0·18, 5·36)		1·22 (0·46, 3·22)
**Primary diverticulitis**				
No	1·00 (reference)			1·00 (reference)
Yes	0·91 (0·27, 3·05)			1·19 (0·42, 3·37)
**Significant interaction terms after backward selection in full factorial analyses**	None	None	None	None

Values in parentheses are 97·5 per cent confidence intervals.

* Cut‐off at optimal sensitivity and specificity according to receiver operating characteristic (ROC) curve analysis. VAS, visual analogue scale; WBC, white blood cell count.

### Reasons for sigmoid resection

Complications of acute diverticulitis (perforation, abscess or bowel obstruction) as the reason for sigmoid resection were mostly comparable between groups (*Table* 6). Outcomes that represent a prolonged or recurrent, but not complicated, disease course (ongoing diverticulitis, persistent abdominal complaints and recurrent diverticulitis) as reason for sigmoid resection appeared to be more common in the observational group.

**Table 6:** Registered reasons for sigmoid resection according to treatment allocation

	Observation	Antibiotics
Diverticular abscess	1	0
Perforated diverticulitis	5	4
Obstruction/chronic ileus	4	3
Ongoing diverticulitis	4	2
Persistent abdominal complaints	5	3
Recurrent diverticulitis	6	2
Diverticular bleeding	1	0
Fistula	1	0
Total	27	14

## Discussion

This individual‐patient data meta‐analysis of two RCTs demonstrated that omitting antibiotics does not increase the risk of ongoing diverticulitis, recurrent diverticulitis, complicated diverticulitis or sigmoid resection. Although some risk factors for adverse events were identified, antibiotic treatment failed to improve outcomes in patients at risk of adverse events.

Earlier results of the DIABOLO^2^ and AVOD^1^ trials showed no significant differences between groups for all outcomes. However, some outcomes at 6 or 12 months showed a trend towards a potential benefit from antibiotics, as suggested by others^6^. These trends could not be confirmed in the present meta‐analysis of 1‐year follow‐up results; there was no statistically significant difference in any outcome between groups. As the present meta‐analysis repeated some analyses from the original study papers, a correction for multiple testing was applied. However, even without this correction the results would have been comparable; only the duration of hospital stay would have been significantly shorter in the observational group in the absence of correction for multiple testing, but the small difference would have made this finding clinically irrelevant. Although not statistically different, the small differences in rates of complicated diverticulitis and sigmoid resection may be considered clinically relevant. The sample size of the meta‐analysis appeared to be insufficient to detect such small differences. However, a statistical power of 80 per cent would need over 2500 patients to test the difference in complicated diverticulitis rates, and over 1800 patients to test the difference in sigmoid resection rates. As AVOD and DIABOLO are the only RCTs available on this topic, it is very unlikely that a sufficient sample size would be achieved.

Uncomplicated acute diverticulitis has been treated routinely with antibiotics for decades, although several guidelines have adapted their recommendations meanwhile. These now state that antibiotics should not be used routinely or can be avoided^7^, ^8^, ^9^, ^10^, ^11^. Some guidelines, however, state that antibiotics ‘should be used selectively’ (American Gastroenterological Association Institute guideline^11^), antibiotics ‘should be given to patients with risk indications of a complicated course’ (German guideline^9^), or antibiotics ‘on a case‐by‐case basis should possibly be considered’ (Italian guideline^8^).

Little is known, however, about risk factors for a complicated course of initially uncomplicated acute diverticulitis. Therefore, guidelines cannot recommend antibiotics for specific patient subgroups, besides subgroups that were excluded from studies on this topic such as immunocompromised patients and women. The present meta‐analysis identified several risk factors for one or more adverse outcomes that can guide the selection of patients who may benefit from antibiotics. However, the results showed that omitting antibiotics does not increase the risk of adverse outcomes in these high‐risk patients. Therefore, prevention of these adverse outcomes with antibiotic treatment may not be warranted.

A strength of this meta‐analysis is the use of individual‐patient data from the only two available RCTs on this topic. A regular meta‐analysis would only have been able to pool results at the (different) follow‐up times reported in the results papers. Furthermore, not all necessary information, about, for instance, the proportion of emergency or elective surgery, was reported in both results papers, and definitions of outcome measures differed between studies. Use of individual‐patient data resolved these issues, and so analyses were more accurate and complete. Although differences between the studies were resolved as much as possible, remaining differences could have influenced the results. Cases of acute diverticulitis within 3 months of randomization were considered ongoing rather than recurrent episodes. In the AVOD trial, however, ongoing diverticulitis was not an outcome measure and recurrent episodes within 3 months were converted into ongoing episodes, even though no data were available to indicate whether patients recovered in between episodes. In addition, to make the trials as homogeneous as possible, all patients with small pericolic abscesses from the DIABOLO trial were excluded. The AVOD trial did not involve such patients, so including these patients with Hinchey 1b disease in the meta‐analysis would not have any added value toward the analyses that have already been performed in the DIABOLO results paper and the number of such patients would still be too small for conclusions to be drawn.

Follow‐up times were made as comparable as possible but some differences probably still exist. The final follow‐up in the AVOD trial took place at different times in the 13th month, whereas in the DIABOLO trial follow‐up during the entire 13th month was available for most patients. Therefore, the follow‐up duration for patients in DIABOLO was slightly longer than that for patients in AVOD. However, if all adverse events from the 13th month in DIABOLO had been excluded (0 cases of complicated diverticulitis; 5 cases of recurrent diverticulitis – 3 in the observational and 2 in the antibiotics group; and 3 sigmoid resections – 2 in the observational and 1 in the antibiotics group), the results would not have changed. Another limitation is the small number of adverse events among patients with acute uncomplicated diverticulitis. Therefore, the statistical power is limited for the analysis of these secondary outcomes, leading to imprecision; this may be a reason for downgrading the level of evidence, as in the American Gastroenterological Association Institute guideline^12^. Finally, both studies excluded patients with sepsis without predefining sepsis, and the AVOD trial lacked a description of patients who were excluded after assessment for eligibility. Although failing to give reasons for exclusion may slightly limit the assessment of generalizability, the fact that only four of 323 patients were excluded owing to sepsis in the DIABOLO trial shows that the effect of this exclusion criterion was most likely minimal.

The decision to treat patients with acute uncomplicated diverticulitis with antibiotics or not does not depend on a single outcome measure. All reasons in favour of using antibiotics and all reasons in favour of omitting them should be taken into account. Omitting antibiotics did not increase the risk of ongoing diverticulitis, recurrent diverticulitis, complicated diverticulitis or sigmoid resection, and is therefore a safe treatment strategy. Insufficient statistical power to detect small differences in complicated diverticulitis and sigmoid resection may leave some room for discussion if these small differences are considered clinically relevant. Furthermore, the use of antibiotics should be limited as much as possible to minimize the global threat of rising microbial resistance and to prevent antibiotic‐related morbidity (8·3 per cent of all patients in DIABOLO^2^), including potentially life‐threatening allergic reactions. In the end, individual‐patient characteristics or preferences may be deciding factors in the decision‐making process. However, in subgroups of patients with independent risk factors for adverse outcome (high pain scores, high WBC and a history of acute diverticulitis at presentation) no beneficial role for antibiotics in the prevention of such adverse outcomes was found.

## Supporting information


**Table S1.** Univariable and multivariable analyses of risk factors (odds ratio) associated with 1 or more episodes of ongoing diverticulitis or complicated diverticulitis, or sigmoid resection.
**Table S2.** Univariable and multivariable analyses of risk factors (odds ratio) associated with 1 or more episodes of ongoing diverticulitis.
**Table S3.** Univariable and multivariable analyses of risk factors (odds ratio) associated with 1 or more episodes of complicated diverticulitis.
**Table S4.** Univariable and multivariable analyses of risk factors (odds ratio) associated with sigmoid resection.
