# Supplementary material for: Observational versus antibiotic treatment for uncomplicated diverticulitis: an individual‐patient data meta‐analysis
Source: Br J Surg. 2020 Feb 19;107(8):1062–9. doi: 10.1002/bjs.11465 (PMC7318319; doi:10.1002/bjs.11465)
Supplement: Supplementary file 1 — Table S1. Univariable and multivariable analyses of risk factors (odds ratio) associated with 1 or more episodes of ongoing diverticulitis or complicated diverticulitis, or sigmoid resection. Table S2. Univariable and multivariable analyses of risk factors (odds ratio) associated with 1 or more episodes of ongoing diverticulitis. Table S3. Univariable and multivariable analyses of risk factors (odds ratio) associated with 1 or more episodes of complicated diverticulitis. Table S4. Univariable and multivariable analyses of risk factors (odds ratio) associated with sigmoid resection. [file BJS-107-1062-s001.docx]

**BJS11465**

**Observational *versus* antibiotic treatment for uncomplicated diverticulitis: an individual-patient data meta-analysis**

**S. T. van Dijk, A. Chabok, M. G. Dijkgraaf, M. A. Boermeester and K. Smedh**

**Table S1 Univariable and multivariable analyses of risk factors (odds ratio) associated with 1 or more episodes of ongoing diverticulitis or complicated diverticulitis, or sigmoid resection.**

|  | No. of patients at risk for ongoing, complicated diverticulitis or sigmoid resection | Ongoing, complicated diverticulitis or sigmoid resection  N (%) | Univariable  (unadjusted for study)  OR (97.5% CI) | Univariable  (adjusted for study)  OR (97.5% CI) | Multivariable  (adjusted for study)  OR (97.5% CI) |
| --- | --- | --- | --- | --- | --- |
| **Sex**  Female  Male | 651  458 | 58 (8.9)  45 (9.8) | 1.11 (0.70-1.78) | 1.08 (0.67-1.73) |  |
| **BMI**  ≤ 30 kg/m^2^  > 30 kg/m^2^ | 636  222 | 65 (10.2)  17 (7.7) | 0.73 (0.39-1.38) | 0.74 (0.39-1.40) |  |
| **Age**  ≤ 50 years  > 50 years | 332  777 | 37 (11.1)  66 (8.5) | 0.74 (0.46-1.20) | 0.74 (0.46-1.21) |  |
| **Comorbidity^†^**  No  Yes | 749  360 | 76 (10.1)  27 (7.5) | 0.72 (0.43-1.21) | 0.66 (0.38-1.13) |  |
| **VAS at presentation^‡^**  ≤ 7  > 7 | 650  261 | 51 (7.8)  37 (14.2) | 1.94 (1.16-3.25) | 1.96 (1.17-3.28) | **1.98 (1.18-3.34)** |
| **Temperature at presentation**  ≤ 38.0 °C  > 38.0 °C | 643  462 | 66 (10.3)  37 (8.0) | 0.76 (0.47-1.23) | 0.81 (0.48-1.37) |  |
| **CRP at presentation^‡^**  ≤ 132 mg/L  > 132 mg/L | 861  247 | 76 (8.8)  27 (10.9) | 1.27 (0.75-2.16) | 1.28 (0.75-2.18) |  |
| **WBC at presentation^‡^**  ≤ 13.5 x10^9^/L  > 13.5 x10^9^/L | 736  370 | 57 (7.7)  45 (12.2) | 1.65 (1.03-2.64) | 1.64 (1.02-2.62) | **1.76 (1.05-2.95)** |
| **Primary diverticulitis**  No  Yes | 247  862 | 32 (13.0)  71 (8.2) | 0.60 (0.36-1.00) | 0.38 (0.19-0.74) | **0.33 (0.16-0.71)** |

^†^ Includes cardiovascular disease and/or pulmonary disease and/or renal failure and/or diabetes mellitus; ^‡^ Cut-off at optimal sensitivity and specificity according to ROC curve analysis

Abbreviations: BMI, Body Mass Index; VAS, Visual Analogue Score; CRP, C-reactive protein; WBC, white blood cell count.

**Table S2 Univariable and multivariable analyses of risk factors (odds ratio) associated with 1 or more episodes of ongoing diverticulitis.**

|  | No. of patients at risk for ongoing diverticulitis | Ongoing diverticulitis  N (%) | Univariable  (unadjusted for study)  OR (97.5% CI) | Univariable  (adjusted for study)  OR (97.5% CI) | Multivariable  (adjusted for study)  OR (97.5% CI) |
| --- | --- | --- | --- | --- | --- |
| **Sex**  Female  Male | 651  458 | 40 (6.1)  27 (5.9) | 0.96 (0.54-1.70) | 0.95 (0.53-1.70) |  |
| **BMI**  ≤ 30 kg/m^2^  > 30 kg/m^2^ | 636  222 | 44 (6.9)  10 (4.5) | 0.64 (0.28-1.42) | 0.62 (0.28-1.40) |  |
| **Age**  ≤ 50 years  > 50 years | 332  777 | 24 (7.2)  43 (5.5) | 0.75 (0.42-1.36) | 0.75 (0.42-1.36) |  |
| **Comorbidity^†^**  No  Yes | 749  360 | 49 (6.5)  19 (5.0) | 0.75 (0.40-1.42) | 0.73 (0.38-1.40) |  |
| **VAS at presentation^‡^**  ≤ 7  > 7 | 650  261 | 37 (5.7)  21 (8.0) | 1.45 (0.77-2.74) | 1.45 (0.77-2.74) |  |
| **Temperature at presentation**  ≤ 38.0 °C  > 38.0 °C | 643  462 | 44 (6.8)  23 (5.0) | 0.71 (0.39-1.29) | 0.69 (0.36-1.30) |  |
| **CRP at presentation^‡^**  ≤ 132 mg/L  > 132 mg/L | 861  247 | 47 (5.5)  20 (8.1) | 1.53 (0.82-2.84) | 1.53 (0.82-2.85) |  |
| **WBC at presentation^‡^**  ≤ 13.5 x10^9^/L  > 13.5 x10^9^/L | 736  370 | 39 (5.3)  28 (7.6) | 1.46 (0.82-2.60) | 1.46 (0.82-2.60) |  |
| **Primary diverticulitis**  No  Yes | 247  862 | 25 (10.1)  42 (4.9) | 0.46 (0.25-0.82) | 0.29 (0.13-0.66) | **0.29 (0.13-0.66)** |

^†^ Includes cardiovascular disease and/or pulmonary disease and/or renal failure and/or diabetes mellitus; ^‡^ Cut-off at optimal sensitivity and specificity according to ROC curve analysis

Abbreviations: BMI, Body Mass Index; VAS, Visual Analogue Score; CRP, C-reactive protein; WBC, white blood cell count.

**Table S3 Univariable and multivariable analyses of risk factors (odds ratio) associated with 1 or more episodes of complicated diverticulitis.**

|  | No. of patients at risk for complicated diverticulitis | Complicated diverticulitis  N (%) | Univariable  (unadjusted for study)  OR (97.5% CI) | Univariable  (adjusted for study)  OR (97.5% CI) | Multivariable  (adjusted for study)  OR (97.5% CI) |
| --- | --- | --- | --- | --- | --- |
| **Sex**  Female  Male | 651  458 | 19 (2.9)  15 (3.3) | 1.13 (0.51-2.47) | 1.19 (0.54-2.63) |  |
| **BMI**  ≤ 30 kg/m^2^  > 30 kg/m^2^ | 636  222 | 19 (3.0)  6 (2.7) | 0.90 (0.31-2.62) | 0.86 (0.30-2.52) |  |
| **Age**  ≤ 50 years  > 50 years | 332  777 | 10 (3.0)  24 (3.1) | 1.03 (0.44-2.42) | 1.02 (0.43-2.41) |  |
| **Comorbidity^†^**  No  Yes | 749  360 | 24 (3.2)  10 (2.8) | 0.86 (0.37-2.03) | 0.95 (0.39-2.28) |  |
| **VAS at presentation^‡^**  ≤ 7  > 7 | 650  261 | 14 (2.2)  15 (5.7) | 2.77 (1.18-6.48) | 2.75 (1.18-6.44) | **2.78 (1.18-6.54)** |
| **Temperature at presentation**  ≤ 38.0 °C  > 38.0 °C | 643  462 | 16 (2.5)  18 (3.9) | 1.59 (0.73-3.48) | 1.46 (0.63-3.40) |  |
| **CRP at presentation^‡^**  ≤ 132 mg/L  > 132 mg/L | 861  247 | 25 (2.9)  9 (3.6) | 1.27 (0.52-3.07) | 1.25 (0.51-3.03) |  |
| **WBC at presentation^‡^**  ≤ 13.5 x10^9^/L  > 13.5 x10^9^/L | 736  370 | 16 (2.2)  17 (4.6) | 2.17 (0.98-4.80) | 2.19 (0.99-4.86) | **2.62 (1.11-6.18)** |
| **Primary diverticulitis**  No  Yes | 247  862 | 12 (4.9)  22 (2.6) | 0.51 (0.23-1.17) | 0.54 (0.20-1.42) |  |

^†^ Includes cardiovascular disease and/or pulmonary disease and/or renal failure and/or diabetes mellitus; ^‡^ Cut-off at optimal sensitivity and specificity according to ROC curve analysis

Abbreviations: BMI, Body Mass Index; VAS, Visual Analogue Score; CRP, C-reactive protein; WBC, white blood cell count.

**Table S4 Univariable and multivariable analyses of risk factors (odds ratio) associated with sigmoid resection.**

|  | No. of patients at risk for sigmoid resection | Sigmoid resection  N (%) | Univariable  (unadjusted for study)  OR (97.5% CI) | Univariable  (adjusted for study)  OR (97.5% CI) | Multivariable  (adjusted for study)  OR (97.5% CI) |
| --- | --- | --- | --- | --- | --- |
| **Sex**  Female  Male | 651  458 | 23 (3.5)  18 (3.9) | 1.12 (0.54-2.29) | 0.96 (0.46-1.98) |  |
| **BMI**  ≤ 30 kg/m^2^  > 30 kg/m^2^ | 636  222 | 28 (4.4)  7 (3.2) | 0.71 (0.27-1.85) | 0.79 (0.30-2.08) |  |
| **Age**  ≤ 50 years  > 50 years | 332  777 | 15 (4.5)  26 (3.3) | 0.73 (0.35-1.54) | 0.74 (0.35-1.57) |  |
| **Comorbidity^†^**  No  Yes | 749  360 | 31 (4.1)  10 (2.8) | 0.66 (0.29-1.52) | 0.48 (0.21-1.12) |  |
| **VAS at presentation^‡^**  ≤ 7  > 7 | 650  261 | 19 (2.9)  16 (6.1) | 2.17 (1.00-4.73) | 2.25 (1.03-4.94) | **2.32 (1.05-5.10)** |
| **Temperature at presentation**  ≤ 38.0 °C  > 38.0 °C | 643  462 | 31 (4.8)  10 (2.2) | 0.44 (0.19-1.00) | 0.65 (0.27-1.57) | 0.66 (0.25-1.70) |
| **CRP at presentation^‡^**  ≤ 132 mg/L  > 132 mg/L | 861  247 | 32 (3.7)  9 (3.6) | 0.98 (0.41-2.32) | 1.02 (0.43-2.43) |  |
| **WBC at presentation^‡^**  ≤ 13.5 x10^9^/L  > 13.5 x10^9^/L | 736  370 | 26 (3.5)  14 (3.8) | 1.07 (0.50-2.29) | 1.03 (0.48-2.21) |  |
| **Primary diverticulitis**  No  Yes | 247  862 | 11 (4.5)  30 (3.5) | 0.77 (0.35-1.73) | 0.06 (0.01-0.60) |  |

^†^ Includes cardiovascular disease and/or pulmonary disease and/or renal failure and/or diabetes mellitus; ^‡^ Cut-off at optimal sensitivity and specificity according to ROC curve analysis

Abbreviations: BMI, Body Mass Index; VAS, Visual Analogue Score; CRP, C-reactive protein; WBC, white blood cell count.
